# Lower loop re‐entrant flutter

**DOI:** 10.1002/joa3.70081

**Published:** 2025-04-30

**Authors:** Anish Bhargav, Ramanathan Velayutham, Raja J. Selvaraj

**Affiliations:** ^1^ Department of Cardiology Jawaharlal Institute of Postgraduate Medical Education and Research Puducherry India

**Keywords:** counterclockwise flutter, crista terminalis, Lower loop reentry

## Abstract

An elderly man with a history of a prior inferior wall myocardial infarction underwent ablation for an atypical right atrial flutter. Electroanatomic mapping revealed diffuse scarring on the anterior, anterolateral, and posterior right atrium, presumably due to atrial infarction from the prior inferior wall myocardial infarction, forcing the activation wavefront through an area of slow conduction across the lower end of the crista terminalis, leading to lower loop reentry in a counterclockwise fashion around the inferior vena cava and a 12‐lead ECG showing positive flutter waves in the inferior leads reflecting septal activation in a cranio‐caudal direction.

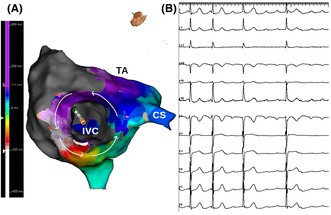

A 55‐year‐old man with a history of a prior inferior wall myocardial infarction underwent ablation for symptomatic, persistent, atypical right atrial flutter. Right atrial mapping was done during flutter, which was persistent with a cycle length of 315 ms. The voltage map showed scar in the anterior, anterolateral, and posterior right atrium with a narrow rim of preserved voltage posteriorly. Activation mapping showed counterclockwise macro‐reentry around the inferior vena cava with breakthrough at the lower end of the crista terminalis. Figure 1, panel A is an inferior view with the inferior vena cava in the center showing the activation wavefront moving in a counterclockwise fashion and encompassing the whole cycle length. Figure 1, panel B is a 12‐lead ECG showing positive flutter waves in the inferior leads largely reflecting septal activation in a cranio‐caudal direction, as there was no activation wavefront over the lateral right atrial wall due to extensive scarring (Figure 2, panel A and B). Entrainment from the mid‐cavotricuspid isthmus showed a post‐pacing interval equal to the tachycardia cycle length with concealed fusion. Linear ablation across the cavotricuspid isthmus resulted in termination of the flutter.

**FIGURE 1:**
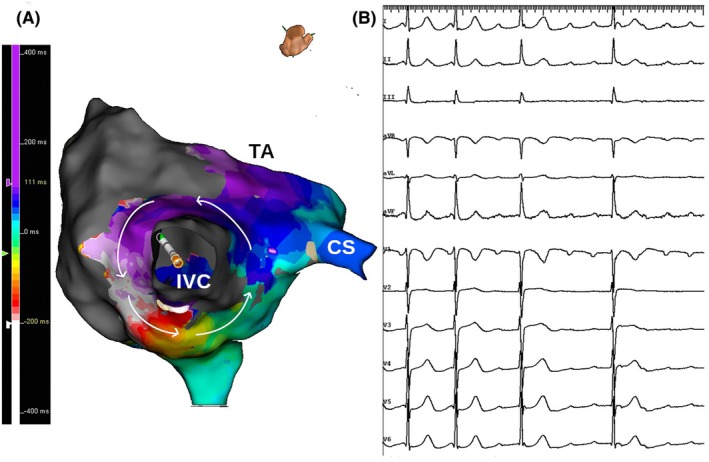
Lower loop reentry. Three‐dimensional electroanatomical activation map of the right atrium in inferior views (Panel A) acquired during right atrial flutter along with 12 lead ECG (Panel B). Panel A highlights the reentry circuit, as delineated by the arrows, with the activation wavefront moving in a counterclockwise fashion and encompassing the whole cycle length. Panel B shows 12 lead ECG with positive flutter waves in inferior leads due to septal activation in a cranio‐caudal direction.

**FIGURE 2:**
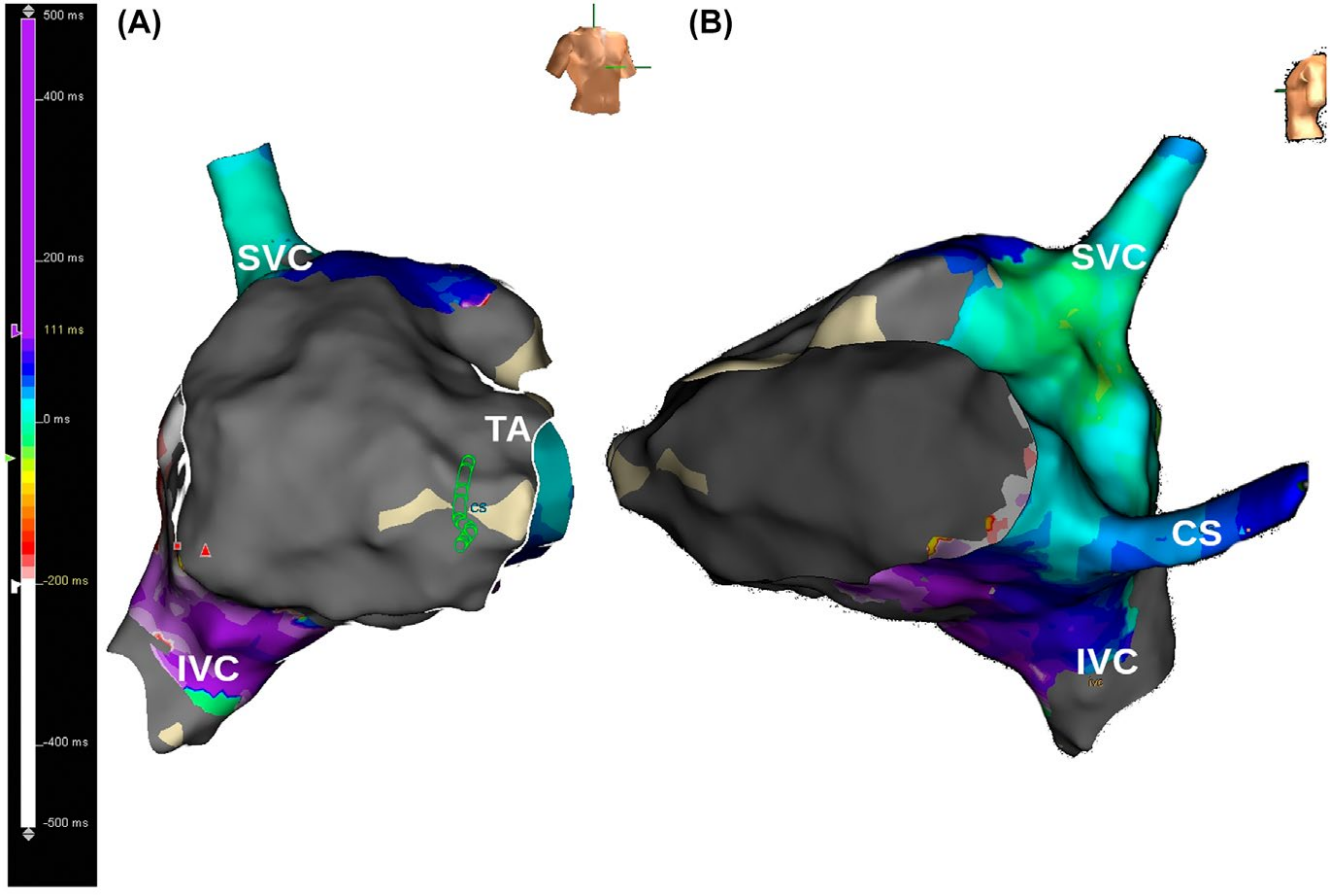
Activation map of the right atrium. Panel A is an RAO view that shows the low voltage area on the anterior, anterolateral, and posterior wall. Panel B is a left lateral view that highlights the cranio‐caudal activation of the atrial septum.

Lower loop re‐entry occurs when an activation wavefront rotates around the inferior vena cava, utilizing the same cavotricuspid isthmus as in typical counterclockwise atrial flutter. It results from a breakdown in the inferoposterior boundaries of the cavotricuspid isthmus formed by the eustachian ridge and lower crista terminalis. This causes the circuit to revolve around the inferior vena cava instead of the tricuspid annulus, across the eustachian ridge through crista terminalis with slow conduction because of transverse activation through crista terminalis. Most of the previously described studies on lower loop re‐entry were predominantly done either without electroanatomic mapping with very few patients of myocardial infarction^1^, ^2^ or with electroanatomic mapping in post‐operative patients with other heart conditions.^3^ In our case, due to the presence of diffuse scar on anterior, anterolateral, and posterior right atrium, presumably due to atrial infarction from prior inferior wall myocardial infarction, the activation wavefront was forced through an area of slow conduction across the lower end of crista terminalis. As it is a cavotricuspid‐dependent macroreentry, linear ablation across isthmus is usually effective.

## CONFLICT OF INTEREST STATEMENT

Authors declare no conflict of interests for this article.

## STATEMENT ON ETHICS AND INTEGRITY POLICIES

I confirm that the current submission is an original work that has not been published previously and is not under review by another publication, and all authors have contributed to the work.

## Data Availability

Available on request.
